# Could Pirfenidone Also be Effective in Treating Intestinal Fibrosis?

**DOI:** 10.3390/cells9081762

**Published:** 2020-07-23

**Authors:** Giovanni Latella, Angelo Viscido

**Affiliations:** Gastroenterology Unit, Department of Life, Health and Environmental Sciences, University of L’Aquila, Piazza S. Tommasi n.1, Coppito, 67100 L’Aquila, Italy; angelo.viscido@univaq.it

Fibrogenesis is a physiological process of tissue repair triggered by acute inflammation, but in chronic inflammation it may become a progressive and independent process leading to fibrosis [1]. Fibrosis is characterized by an excessive local accumulation of extracellular matrix (ECM) proteins (mainly collagen), due to an increase in their production by activated fibroblasts and myofibroblasts and/or the reduction in their degradation by specific matrix metalloproteinases (MMPs).

Intestinal fibrosis is a common outcome in inflammatory bowel diseases (IBD), especially in Crohn’s disease (CD) in which it becomes clinically apparent in up to 40% of patients. In CD, fibrosis may lead to intestinal fibrostrictures and even obstruction, that requires surgery [2]. Intestinal fibrostrictures are currently treated by endoscopic pneumatic dilations and surgical procedures, but even after surgery, there is a high rate of recurrence often leading to further surgical procedures. The cellular and molecular mechanisms underlying the formation of intestinal fibrostrictures in CD are not understood. Currently, effective pharmacological treatments to control or reverse the fibrosis process are unavailable.

Several putative antifibrotic compounds have been tested, but only pirfenidone has recently been approved for the treatment of pulmonary fibrosis [3]. Therefore, it has been suggested that pirfenidone could also be effective in the treatment of intestinal fibrosis.

In a recent issue of *Cells*, Cui Y et al. have evaluated the anti-proliferative and anti-fibrotic properties of pirfenidone on primary human intestinal fibroblasts (p-HIFs) [4]. Pirfenidone dose-dependently inhibited p-HIF proliferation and motility, without inducing cell death, a reversible inhibition. Furthermore, Pirfenidone reduced mRNA levels of gene encoding ECM proteins like COL1A1, COL3A1, COL4A1, COL6A1, FN1 (encoding fibronectin 1) and ELN (encoding elastin), while it did not affect ACTA2 (encoding alpha-smooth muscle actin: α-SMA considered a marker of activated myofibroblasts). Pirfenidone also suppressed basal and TGF-β1-induced collagen I expression in p-HIFs. Pirfenidone inhibited the proliferation of intestinal fibroblasts and their production of the collagen I by suppressing both basal and TGF-β1-induced phosphorylation of mTOR and p70S6K but without affecting the phosphorylation of SMAD2/3 and p38 MAPK.

These results are of great interest but should be confirmed on human intestinal fibroblasts isolated from both the intestines of healthy subjects and CD patients. In particular, they should be confirmed both on fibroblasts from the mucosa of the colon and the terminal ileum, the latter being the segment most frequently affected by fibrostrictures in CD. The effects of Pirfenidone reported on fibroblasts from a normal colon could not be confirmed on fibroblasts from CD fibrostrictures. 

Kadir et al. reported that Pirfenidone inhibited the proliferation of fibroblasts from patients with active CD and the production of MMP3 and increased αSMA expression but did not influence neither the tissue inhibitor of metalloproteinases-1 (TIMP-1), nor collagen production [5].

Inhibition of intestinal fibroblast proliferation by pirfenidone has been reported in several experimental studies both in vitro and in vivo [6,7,8,9,10,11]. Pirfenidone has been shown to induce intestinal fibroblast apoptosis and to inhibit TGF-β1-induced HIF activities such as myofibroblast differentiation (α-SMA) and collagen production [7,8,9,10]. These effects of Pirfenidone were mediated by the inhibition of several signalings, including Smad, PI3K/AKT, MAPK, and mTOR transduction pathways [7,8,9,10]. Furthermore, pirfenidone can also act on heat shock protein 47 (Hsp47), plasminogen activator inhibitor-1 (Pai-1), platelet-derived growth factors (PDGF), connective tissue growth factors (CTGF), and transient receptor potential ankyrin 1 (TRPA1) channel pathways [6,8,9]. The discrepancies reported by the various studies on the effects of pirfenidone on these molecular pathways need to be clarified.

TGF-β1 represents the main driving force of fibrosis in numerous organs including the intestine. The pivotal pro-fibrogenic role of TGF-β1 can be achieved through the activation of different intracellular signalings, including SMAD, MAPK and mTOR pathways [1]. However, it has not yet been established which of these TGF-β1 transduction pathways plays a major role in the modulation of inflammation and which in the modulation of fibrogenesis.

The initiation, maintenance, and progression of intestinal fibrosis are probably linked to the persistence of a possible pro-fibrogenic adaptive immune response by specific T helper (Th) lymphocytes that trigger and maintain active the pro-fibrogenic functions of mesenchymal stromal cells (fibroblasts, myofibroblasts, pericytes, endothelial cells). Therefore, it would be very important to evaluate also the effect of Pirfenidone on the activation, polarization, and proliferation of the various Th subsets (Th1, Th2, Th9, Th17, Th22, Treg cells), some with pro-fibrogenic action others with anti-fibrogenic effects [12]. Inhibition of pro-fibrogenic Th subsets could certainly reduce or even shut down the pro-fibrogenic activities of intestinal fibroblasts and myofibroblasts. 

The treatment with pirfenidone is often associated with gastrointestinal side effects, including dyspepsia, nausea, vomiting, abdominal pain and diarrhea. To avoid or at least minimize these gastrointestinal side effects of Pirfenidone, which could be even more frequent and severe in CD, Cui et al. also suggest for pirfenidone the use of controlled intestinal release systems of the drug similar to as was done with mesalazine. Unfortunately, this hypothesis would be limited by the fact that pirfenidone demonstrated a poor topical efficacy [10]. 

Some cases of colitis associated with the use of pirfenidone in pulmonary fibrosis were also reported [13]. Pirfenidone could have negative effects on intestinal epithelial cells (reducing their proliferation, altering their junction proteins, inducing apoptosis) and therefore could alter the permeability and the barrier function of the intestinal mucosa. The loss of the barrier function can lead to translocation in the mucosa of microbes, whose components or products continue to stimulate the local immune response responsible for chronic intestinal inflammation and related fibrosis. Therefore, in addition to the effects on the pro-fibrogenic functions of fibroblasts, it is also crucial to evaluate the effects of pirfenidone on intestinal epithelial cells.

Furthermore, it would be useful to evaluate whether the observed anti-fibrotic effects of pirfenidone are entirely direct effects or partly secondary to its anti-inflammatory and anti-oxidant actions.

In conclusion, pirfenidone has important therapeutic potential to be used as an anti-inflammatory and anti-fibrotic agent also in IBD, especially in CD. However, further studies are needed to evaluate its effects not only on mesenchymal stromal cells, such as fibroblasts and myofibroblasts but also on immune and epithelial cells, both in vitro experimental models and experimental models of chronic colitis complicated by intestinal fibrosis [6,8,10,11].

## References

[1] Latella G., Di Gregorio J., Flati V., Rieder F., Lawrance I.C. (2015). Mechanisms of initiation and progression of intestinal fibrosis in IBD. Scand J. Gastroenterol..

[2] Rieder F., Latella G., Magro F., Yuksel E.S., Higgins P.D., Di Sabatino A., de Bruyn J.R., Rimola J., Brito J., Bettenworth D. (2016). European Crohn’s and Colitis Organisation Topical Review on Prediction, Diagnosis and Management of Fibrostenosing Crohn’s Disease. J. Crohns Colitis.

[3] Nathan S.D., Costabel U., Glaspole I., Glassberg M.K., Lancaster L.H., Lederer D.J., Pereira C.A., Trzaskoma B., Morgenthien E.A., Limb S.L. (2019). Efficacy of Pirfenidone in the Context of Multiple Disease Progression Events in Patients With Idiopathic Pulmonary Fibrosis. Chest.

[4] Cui Y., Zhang M., Leng C., Blokzijl T., Jansen B.H., Dijkstra G., Faber K.N. (2020). Pirfenidone Inhibits Cell Proliferation and Collagen I Production of Primary Human Intestinal Fibroblasts. Cells.

[5] Kadir S.I., Wenzel Kragstrup T., Dige A., Kok Jensen S., Dahlerup J.F., Kelsen J. (2016). Pirfenidone inhibits the proliferation of fibroblasts from patients with active Crohn’s disease. Scand. J. Gastroenterol..

[6] Iswandana R., Pham B.T., Suriguga S., Luangmonkong T., van Wijk L.A., Jansen Y.J.M., Oosterhuis D., Mutsaers H.A.M., Olinga P. (2020). Murine Precision-cut Intestinal Slices as a Potential Screening Tool for Antifibrotic Drugs. Inflamm. Bowel Dis..

[7] Sun Y., Zhang Y., Chi P. (2018). Pirfenidone suppresses TGF-β1-induced human intestinal fibroblasts activities by regulating proliferation and apoptosis via the inhibition of the Smad and PI3K/AKT signaling pathway. Mol. Med. Rep..

[8] Kurahara L.H., Hiraishi K., Hu Y., Koga K., Onitsuka M., Doi M., Aoyagi K., Takedatsu H., Kojima D., Fujihara Y. (2017). Activation of Myofibroblast TRPA1 by Steroids and Pirfenidone Ameliorates Fibrosis in Experimental Crohn’s Disease. Cell. Mol. Gastroenterol. Hepatol..

[9] Sun Y.W., Zhang Y.Y., Ke X.J., Wu X.J., Chen Z.F., Chi P. (2018). Pirfenidone prevents radiation-induced intestinal fibrosis in rats by inhibiting fibroblast proliferation and differentiation and suppressing the TGF-β1/Smad/CTGF signaling pathway. Eur. J. Pharmacol..

[10] Li G., Ren J., Hu Q., Deng Y., Chen G., Guo K., Li R., Li Y., Wu L., Wang G. (2016). Oral pirfenidone protects against fibrosis by inhibiting fibroblast proliferation and TGF-β signaling in a murine colitis model. Biochem. Pharmacol..

[11] Meier R., Lutz C., Cosín-Roger J., Fagagnini S., Bollmann G., Hünerwadel A., Mamie C., Lang S., Tchouboukov A., Weber F.E. (2016). Decreased Fibrogenesis After Treatment with Pirfenidone in a Newly Developed Mouse Model of Intestinal Fibrosis. Inflamm. Bowel. Dis..

[12] Latella G., Viscido A. (2020). Controversial Contribution of Th17/IL-17 toward the Immune Response in Intestinal Fibrosis. Dig. Dis. Sci..

[13] Fiddler C.A., Simler N., Parfrey H., Miremadi A., Chilvers E.R. (2016). Severe Colitis Associated with Pirfenidone Use in Idiopathic Pulmonary Fibrosis. Ann. Am. Thorac. Soc..

